# Studying the sense of agency in the absence of motor movement: an investigation into temporal binding of tactile sensations and auditory effects

**DOI:** 10.1007/s00221-021-06087-8

**Published:** 2021-04-07

**Authors:** S. Antusch, R. Custers, H. Marien, H. Aarts

**Affiliations:** Department of Psychology, Utrecht University, PO BOX 80140, 3508 TC Utrecht, The Netherlands

**Keywords:** Sense of agency, Tactile input, Temporal binding, Intentional action

## Abstract

People form coherent representations of goal-directed actions. Such agency experiences of intentional action are reflected by a shift in temporal perception: self-generated motor movements and subsequent sensory effects are perceived to occur closer together in time—a phenomenon termed intentional binding. Building on recent research suggesting that temporal binding occurs without intentionally performing actions, we further examined whether such perceptual compression occurs when motor action is fully absent. In three experiments, we used a novel sensory-based adaptation of the Libet clock paradigm to assess how a brief tactile sensation on the index finger and a resulting auditory stimulus perceptually bind together in time. Findings revealed robust temporal repulsion (instead of binding) between tactile sensation and auditory effect. Temporal repulsion was attenuated when participants could anticipate the identity and temporal onset (two crucial components of intentional action) of the tactile sensation. These findings are briefly discussed in the context of differences between intentional movement and anticipated bodily sensations in shaping action coherence and agentic experiences.

**Supplementary Information:**

The online version contains supplementary material available at 10.1007/s00221-021-06087-8.

Individuals continuously engage in intentional behavior to control and shape the external world. They select and time their courses of action based on anticipated outcomes and act accordingly. Apart from preparing and executing actions intentionally, humans also form coherent representations of their own behavior (Aarts and Custers 2009; Vallacher and Wegner 1987). Planning actions and causing effects, such as pressing keys that result in a sound or light, enables their perception as coherent events of meaningful behavior (e.g., playing a tone on the piano or illumining a room). An important contribution to the study of conscious experiences of action coherence comes from research on temporal binding (Haggard et al. 2002a, b). Intentional actions and their effects are bound together in temporal perception—a phenomenon coined intentional binding. Intentional binding is believed to be intimately linked to the sense of agency, and thus essential for attributing behavior to the self and arriving at feelings of responsibility (Moore and Obhi 2012).

While intentional binding is a widely replicated and robust effect, the exact role of intentions remains uncertain, as does the link with sense of agency. Primary support for the link with agency experiences comes from studies using transcranial magnetic stimulation (TMS), showing that abrupt stimulation of cortical motor areas results in temporal repulsion instead of temporal binding between the induced action and a subsequent effect (Haggard and Clark 2003; Haggard et al. 2003). Interestingly, TMS studies are among the only studies showing this strong repulsion effect for involuntary actions. In situations in which a person’s action is passively induced by other external means, such as a mechanical lever press, temporal binding, though less strong, still emerges (Desantis et al. 2011; Dogge et al. 2012; Kirsch et al. 2019). Furthermore, it even persists when individuals observe intentional actions in (virtual) others (Suzuki et al. 2019; Vastano et al. 2018). These findings suggest that not intentionality per se, but other processes that are associated with intentional action, such as knowledge about causality and predictions that one can make based on this knowledge, may drive temporal binding (Buehner and Humphreys 2010; Desantis et al. 2011).

It is important to note that in passive movement studies, crucial components of intentional action are not entirely ruled out (Antusch et al. 2020). Participants can mentally simulate their action, or even start up their motor system once they notice a slight movement induced by a mechanical lever, allowing them to time and predict the effect resulting from their key press. Because of the tight link between perception and action (e.g., Decety and Grèzes 2006; James 1890; Prinz 1997), a similar reasoning might explain temporal binding effects as a result of observing others’ action.

In the present research we further explore temporal binding in the absence of action, rendering an explanation in terms of motor simulation unlikely. We took on a novel methodological approach that abolishes the action component completely and allows for the examination of temporal binding between a key sensation of action movement and an effect. That is, using a version of the Libet clock intentional binding task (Haggard et al. 2002a, b), that successfully and consistently produced binding effects in our lab (Antusch et al. 2019, 2021) we tested whether and when induced tactile sensation in the fingertips, i.e., the sensory input that commonly accompanies key presses, is temporally bound to auditory effects. If temporal binding also occurs between a tactile (fingertip) sensation and a resulting auditory stimulus, this would suggest that simulating and executing a motor movement is not crucial for its emergence. In return, this would also cast doubt on the role of temporal binding as a reflection of a sense of agency.

Recently, research started to explore whether temporal binding between body sensations can occur in the absence of any motor movement. Using a stringent and radical test and employing a single-modal sensory paradigm that rules out any effect of motor-related activity, Antusch and colleagues (2020) examined whether an auditory stimulus (brief presentation of a noise) and another subsequent auditory stimulus (a sinus tone) are perceptually bound together in time. Instead of being experienced as attracted to each other, as is the case in temporal binding, a clear temporal repulsion effect was found. That is, the auditory stimuli were experienced as shifted away from each other (cf. Desantis et al. 2012; Haggard et al. 2002a). Importantly, inducing beliefs about causality as well as prior learning of causality between the stimuli did not transform temporal repulsion into binding.

One possible explanation as to why two causally related auditory events are repulsed from each other in temporal perception might be that the predictive auditory cue is exogenous to the agent, thereby reducing the perceived coherence between the two contingent auditory stimuli. It is conceivable that for temporal binding between causally related sensations to occur, an agent needs to process sensory information that usually accompanies endogenous motor movement and is crucial for the feeling of being an intentional agent. An important type of such information is tactile sensation. For example, a finger press on a key not only causes an auditory effect (e.g., an auditory stimulus) but also changes the tactile sensation in the fingertip stemming from the press itself. This movement-related haptic input of self-selected action is discrete and dissimilar from sensory input of the auditory effect that is caused by the action (Aschersleben and Prinz 1995; Mates et al. 1992).

Evidence for the importance of tactile sensation in facilitating temporal binding and agency experiences comes from recent research that examined the effects of tactile sensation as part of motor movement. In this research, participants had to produce an effect (e.g., tone) by “touching” their skin or a mid-air interface (e.g., pushing a virtual visual button and receiving haptic feedback through a vibrating glove or ultrasound pressure waves), thus receiving tactile input followed by the effect. This tactile or mid-air feedback, when compared to conventional button presses or touchpad input, seems to increase sense of agency and temporal binding between tactile stimulus and effect (Bergstrom-Lehtovirta et al. 2018; Cornelio-Martinez et al. 2018; Coyle et al. 2012). Whereas informative, these studies do not directly examine whether tactile sensation in itself enhances temporal binding and the sense of agency. Specifically, they allowed participants to intentionally move their finger, thus confounding intentional action and tactile sensation in the absence of action. Accordingly, it remains an open question whether temporal binding between action and effect requires agents to intentionally simulate and actually execute motor movement, or whether tactile sensation suffices for shaping a sense of agency.

Here, we examined the role of sheer tactile stimulation in the sense of agency by systematically testing whether tactile feedback and a subsequent auditory effect are perceptually bound together in time. To assess temporal binding in a context where motor action is entirely absent, we applied a sensory-based adapted Libet clock paradigm that allowed us to measure the subjective compression of the time-interval between tactile sensation and a subsequent auditory effect. Tactile sensation was delivered to a participant’s left or right index finger, followed by a high or low frequency tone. The mapping of the tones onto the two fingers was counterbalanced between participants but followed a 100% contingency. Thus, the task allowed for specific predictions of action-outcomes, which may promote sense of agency (Dogge et al. 2019a). Experiment 1 was designed to test the basic effect of our paradigm. Against the backdrop of earlier research on auditory (single-modality) sensation and movement-related tactile sensation (Antusch et al. 2020; Bergstrom-Lehtovirta et al. 2018), both—temporal repulsion or binding—were conceivable findings. Importantly, intentional action is assumed to originate from people’s ability to determine the identity and temporal onset of motor movement, that is which action will be selected at which moment in time (Brass and Haggard 2008). Taking these crucial agency properties of intentional action into account, we examined the identity feature by explicitly informing participants which tactile fingertip sensation (i.e., either on the left or the right) would occur (Experiment 2), and tested the temporal onset feature by instructing them to carefully use a countdown procedure to anticipate the temporal onset of the tactile fingertip sensation (Experiment 3). In line with earlier research, this agentic information might facilitate temporal binding.

## Methods

### Participants and design

Twenty-seven^1^ (21 females) volunteers with a mean age of 22 years (*M* = 22.3, SD = 2.98) took part in the experiment for course credit or a monetary reimbursement. All participants gave written informed consent.

The experiment used a 2 (target: tactile vs. auditory stimulus) × 2 (type of trial: baseline vs. succession trials) within-subjects design. All experiments presented in this article were programmed in E-prime Software version 2.0 and received approval from the faculty’s Ethics Review Board (ethics approval code: FETC17-124).

### Stimuli and procedure

Stimuli: Four different stimuli of 100 ms duration each were used: (1) a tactile sensation on the left index finger, (2) a tactile sensation on the right index finger, (3) a low tone of 300 Hz and (4) a high tone of 1000 Hz frequency. The sinusoidal tones were played via a Sennheiser headphone. The tactile sensations were small vibrations caused by a 50 Hz sinusoidal signal (inaudible through the headphones) played via an audio exciter (TEAX14C02-8 Compact Audio Exciter, Tectonic Elements) that was mounted on one cm thick wooden boards.

Acquisition phase: Participants were seated in front of a computer screen in a cubicle. To assure adherence to the instructions, the experimenter remained in the cubicle for the duration of the experiment. All instructions were provided on screen.

**Fig. 1:**
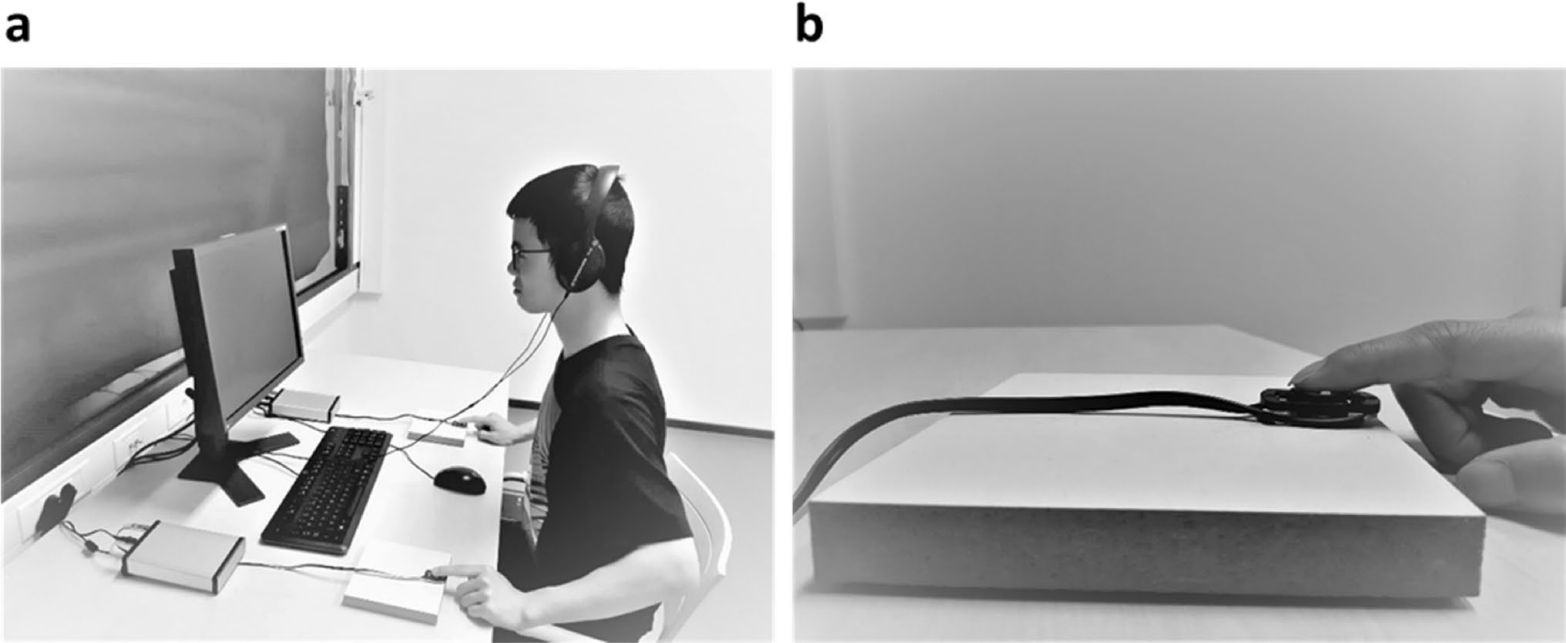
Experimental set-up in the cubicle (**a**); apparatus via which the tactile sensation was delivered (**b**)

Participants put on the headphone and placed their index fingers on the apparatuses positioned right and left of the keyboard (see Fig. 1 for the experimental set-up). To acquaint them with the stimuli, participants first experienced all four stimuli separately in 24 randomized trials. Next, they learned that each tactile sensation would result in one of the auditory stimuli. The two tactile sensation—auditory stimulus pairings (i.e., left tactile + 300 Hz & right tactile + 1000 Hz and left tactile + 1000 Hz & right tactile + 300 Hz) were counterbalanced in order and across participants but remained constant per participant. Hence, each participant learned two stimuli pairs which were presented five times each in randomized order (i.e., 10 trials in total). Participants were asked to pay close attention to the pairs.

Afterwards, participants were presented with the two learned and two novel stimuli pairs, formed by crossing the combinations (i.e., tactile sensations were presented with the auditory stimulus of the respective other stimuli pair) and asked to correctly identify the learned pairs and negate the novel pairs.

Experimental trials (altered Libet clock trials): The experimental sensory-based adapted Libet clock task resembled those commonly used to assess intentional binding (e.g., Haggard et al. 2002a). Participants completed four randomized blocks: a baseline tactile block, a baseline auditory block, a succession tactile block and a succession auditory block. A block consisted of 25 trials (five practice trials and 20 experimental trials), resulting in a total of 100 trials. Practice trials were indistinguishable from experimental trials but were not analyzed.

Each trial began with the presentation of a clock face with a rotating clock hand. The clock face had a diameter of six cm and comprised 40 grey dots arranged in a circle from the center of the screen. A black dot that moved at a period of 2560 ms served as the clock hand. Per trial, participants received either one stimulus (tactile sensation or tone) or two stimuli in succession (tactile sensation and tone). In baseline blocks, participants experienced either a tactile sensation (baseline tactile blocks) or a tone (baseline tone blocks). In succession blocks, participants experienced a tactile sensation followed by the respective paired tone 250 ms later. The temporal onset of the tactile sensation (or the tone in the baseline tone trials) was programmed to occur at a random moment during the second rotation of the clock hand. After the last stimulus was presented, the clock hand rotated further and disappeared between 1000 and 2000 ms later.

**Fig. 2:**
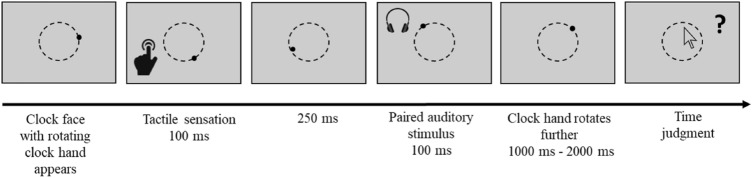
Schematic overview of succession trials in Experiment 1. Baseline trials only included the presentation of a single stimulus (either tactile or auditory)

Participants’ task was to judge the temporal onset of the tactile stimulation or the auditory (tone) stimulus. Participants estimated the temporal onset of a tactile sensation in baseline tactile and half of tactile—tone trials (i.e., succession tactile trials), whereas they estimated the temporal onset of a tone in baseline tone and half of the tactile—tone trials (i.e., succession tone trials). They judged the perceived onset of the respective stimulus by clicking on one of the dots of the clock face with the mouse cursor. Judgment errors denoting the difference between the actual onset of the stimulus and the estimated onset in milliseconds were logged. The inter-trial interval was 1000 ms. See Fig. 2 for a schematic overview of succession trials.

Finally, participants reported their age and gender, were thanked for their participation, reimbursed and debriefed.

### Data analysis plan

The data of five participants were excluded because they failed to correctly identify the learned stimuli pairs, resulting in a final sample of 22 participants. Next, for each participant, mean judgment errors per block and separate shifts for the tactile and auditory stimulus were computed. Extreme judgment errors more than ± 640 ms away from the actual temporal onset were excluded as inattentiveness on those trials can be assumed (Aarts et al. 2012). This resulted in the exclusion of 0.19% of all trials. Perceptual shifts were calculated by subtracting the mean judgment error of baseline trials from the mean judgment error of succession trials (tactile shift = mean judgment error of succession tactile trials—mean judgment error of baseline tactile trials; tone shift = mean judgment error of succession tone trials—mean judgment error of baseline tone trials). Finally, overall binding scores were computed by subtracting the shift of the second stimulus from the shift of the first stimulus (i.e., overall score = tactile shift—tone shift). Positive overall binding scores thus indicated temporal compression of the interval between the tactile and auditory stimuli (temporal binding) while negative scores were indicative of temporal repulsion (see Supplementary Materials for further details).

Next, we continued with the main analysis and subjected the overall binding scores to a one-sample *t*-test against zero to assess the direction of the effect (i.e., temporal binding or temporal repulsion). Based on the literature either temporal binding or temporal repulsion were possible findings (Antusch et al. 2020; Bergstrom-Lehtovirta et al. 2018) and since we had no clear hypothesis as to the direction of the effect, we decided for a two-tailed test.

Additionally, and to illustrate the strength of the evidence, parallel Bayesian analyses were performed. For the convenience of the reader, complete Bayesian analyses are reported*.*

## Results

**Fig. 3:**
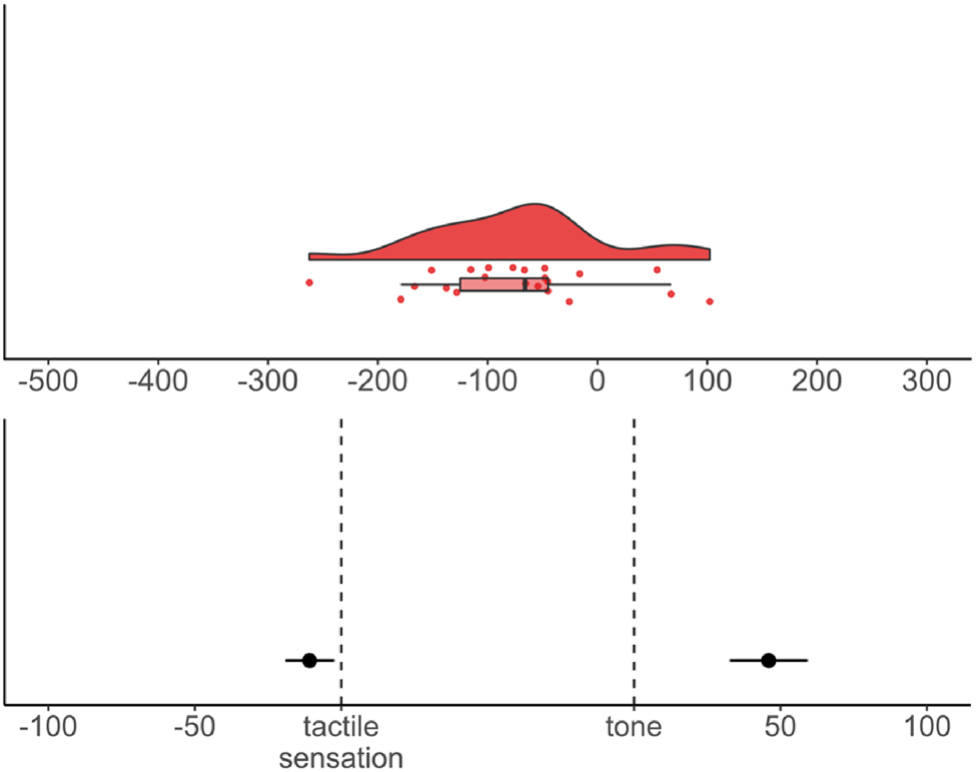
Top: Distribution of the overall temporal binding score in ms (positive scores indicate binding while negative scores indicate temporal repulsion), dots show the distribution of the individual data points and boxplot summaries use the median. Bottom: Separate temporal shifts from baseline in ms, dots denote the mean, bars represent standard errors

Overall binding: A two-tailed one-sample *t*-test on the overall shift score (*M* = − 73.13, SD = 83.93) against zero was conducted. Results revealed a statistically significant temporal repulsion effect, *t*(21) =  − 4.09, *p* = 0.001, *d* = − 0.87, 95% CI [− 1.36, − 0.37]. Visualizations of the distribution of the overall temporal binding scores and the shifts are provided in Fig. 3.

### Bayesian analysis

Bayesian analysis for all experiments were performed in JASP (JASP Version 0.9, JASP Team 2018). As the determination an informed prior was difficult, the default Cauchy prior *r* = 0.707 was used. Robustness checks are reported in the Supplementary Materials. Bayes factors are always reported testing the alternative hypothesis against the null hypothesis (two-tailed tests: BF_10,_ one-tailed tests: BF_+0_), credibility intervals are provided.

Overall binding: A Bayesian one-sample *t*-test testing the null hypothesis that the population mean was equal to zero against the alternative hypothesis that the population mean was not equal to zero was conducted. The test revealed a BF_10_ = 62.69, 95% CrI [− 1.3, − 0.31], indicating that the data was 62 times more likely to occur under the alternative than under the null hypothesis. Hence, there was very strong evidence in favor of the alternative hypothesis.

### Experiment 2

Experiment 1 showed that a tactile sensation and a resulting tone are shifted away from each other in temporal perception, paralleling a temporal repulsion effect. It is important to note that participants directly received a tactile sensation on the left or right index finger without any prior knowledge about which finger would be stimulated. Intentional actions, however, are carried out in line with goals (e.g., pushing left causes a high tone), informing the agent which action is selected. In Experiment 2, we therefore included cues at 550 ms before the tactile sensation (i.e., the lag with which intentions in voluntary action are thought to precede actions; Libet et al. 1983), indicating to participants which tactile sensation would occur. These cues thus allowed participants to anticipate the identity of the sensation (left or right). If such identify information is central to intentional action and important for the sense of agency, temporal repulsion might decrease or even be reversed, resulting in temporal binding when participants know on which index finger the tactile sensation will occur.

## Methods

### Participants and design

Thirty-seven^2^ (24 females) new volunteers with a mean age of 24 years (*M* = 23.8, SD = 4.51) participated in the study in exchange for course credit or monetary reimbursement. All participants gave written informed consent.

We slightly changed the experimental procedure of Experiment 1 to render the control condition comparable to the new identity cue condition (see below). Therefore, for the sake of proper testing, in Experiment 2 we compared the control condition with an identity cue condition in a within-subjects task setting. Specifically, we used a 2 (stimulus: tactile vs. auditory) × 2 (type of trial: baseline vs. succession) × 2 (condition: control vs. identity) within-subjects design.

### Procedure

The general procedure and the experimental task resembled Experiment 1. The stimuli were identical. Differences between the experiments are outlined below.

Acquisition phase*:* Participants completed the same acquisition phase as in Experiment 1. However, to avoid exclusion of participants (see Experiment 1), participants only advanced to the experimental trials after correctly identifying the learned and negating the novel tactile-auditory pairs and were otherwise redirected to the learning trials.

Experimental trials (altered Libet clock trials): In Experiment 2, a second within-subjects condition was added. Hence, participants completed two conditions in counterbalanced order: a *no cue control condition* (same as in Experiment 1) and an *identity cue condition*.

Both conditions consisted of the same four randomized blocks of experimental trials. To familiarize participants with the differences between the conditions, they completed four separate learning trials at the beginning of each condition—one of each type of block (i.e., baseline tactile, a baseline auditory, a succession tactile and a succession auditory trial in sequential order).

**Fig. 4:**
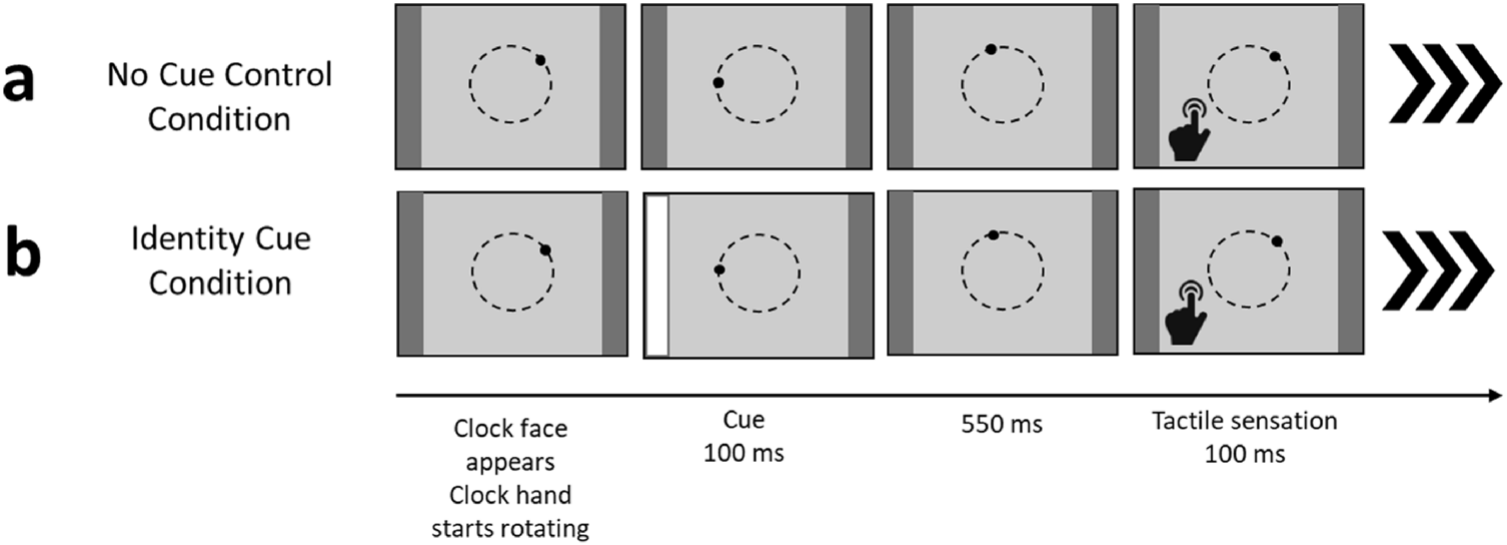
Schematic overview of the start of a trial in two types of conditions: **a** no cue control condition (identical to Exp. 1) and **b** the identity cue condition. Identity cues (left or right flash) occurred at a random moment during the second rotation of the clock, followed by the corresponding (left or right) tactile stimulation. In succession trials, tactile stimulation was followed by a tone. In baseline trials, only tactile stimulation or a tone was presented. The triple arrows (in bold) indicate the remaining part of a trial, which was identical to Experiment 1

Contrary to Experiment 1, the clock face was framed by two vertical grey columns on the left and the right side of the screen. This was done to integrate the visual cue in the Libet clock task. In the *no cue control condition*, these columns always remained grey (see Fig. 4). In the *identity cue condition*, one of the columns (left or right) randomly flashed up in white for 100 ms. A left flash indicated a tactile sensation on the left index finger, and a right flash a tactile sensation on the right index finger. The flash occurred at a random moment during the second rotation of the clock hand. Thus, participants were not able to anticipate the temporal onset of the flash. They were told to attend to the identity of the flash (left or right), as this would inform them which index finger would receive tactile stimulation. On baseline auditory trials, no cue was presented because no tactile sensation occurred on those trials. A visualization of the start of a trial is given in Fig. 4.

While earlier research showed robust binding effects for fewer trials (Cornelio Martinez et al. 2017, study 1; Moore et al. 2010), in Experiment 2, we increased the number of test trials per block from 20 to 30 (excluding four practice trials) to better control for the influence of excluded or extreme temporal estimates. That is, each participant completed 272 trials in total.

### Data analysis plan

As before, extreme judgment errors more than ± 640 ms away from the actual temporal onset were excluded as inattentiveness on those trials can be assumed (Aarts et al. 2012). This resulted in the exclusion of 0.7% of all trials. Next, perceptual shift scores for both stimuli (i.e., tactile sensation and auditory tone) as well as mean binding scores in both conditions (i.e., no cue control condition and the identity cue condition) and overall binding scores were calculated for each participant (see Experiment 1 for the exact formulas).

Before the main analysis, we assured that the order in which the conditions were completed did not affect the results. To this end, a repeated measures ANOVA with cue as a within-subjects factor and order as a between-subjects factor was conducted. Next and after collapsing the data, we subjected the overall binding scores to a two-tailed one-sample *t-*test against zero to test whether the temporal repulsion effect of Experiment 1 was replicated or reversed into binding. Since we added a new condition and only had one prior result, we decided for a two-tailed test as we did not have a clear expectation about the direction (i.e. temporal binding or repulsion) of the effect. Then we conducted a one-tailed paired samples *t*-test testing our hypothesis that the identity cue attenuates temporal repulsion (indicative of more coherence perception). Finally, we assessed whether temporal binding or repulsion was present in both conditions. Hence, we conducted two-tailed one-sample *t*-tests against zero using the overall binding scores in the respective conditions.

Additionally, equivalent Bayesian analyses were conducted.

## Results

Effect of order: The repeated measures ANOVA with cue as a within-subjects factor and order as a between-subjects factor showed that neither the main effect of cue, *F*(1,35) = 3.68, *p* = 0.063, *η*_*p*_^2^ = 0.10, the main effect of order, *F*(1,35) = 0.01, *p* = 0.946, *η*_*p*_^2^ = 0.00, nor the interaction between cue and order, *F*(1,35) = 1.52, *p* = 0.226, *η*_*p*_^2^ = 0.04, was significant and the data was therefore collapsed.

Overall binding: Subjecting the overall binding score to a two-tailed one-sample *t*-test against zero showed that the overall temporal repulsion effect (*M* = − 34.78, SD = 116.54) was not significantly different from zero, *t*(36) =  − 1.82, *p* = 0.078, *d* = − 0.32, 95% CI [− 0.65, 0.01].

Influence of cue: Results of the one-tailed paired samples *t*-test confirmed that temporal repulsion in the identity cue condition (*M* = − 19.41, SD = 129.31) was significantly smaller than in the no cue control condition (*M* = − 50.15, SD = 122.89), *t*(36) =  − 1.94, *p* = 0.031, *d*_*z*_ = − 0.24, 95% CI [− 0.5, 0.01].

**Fig. 5:**
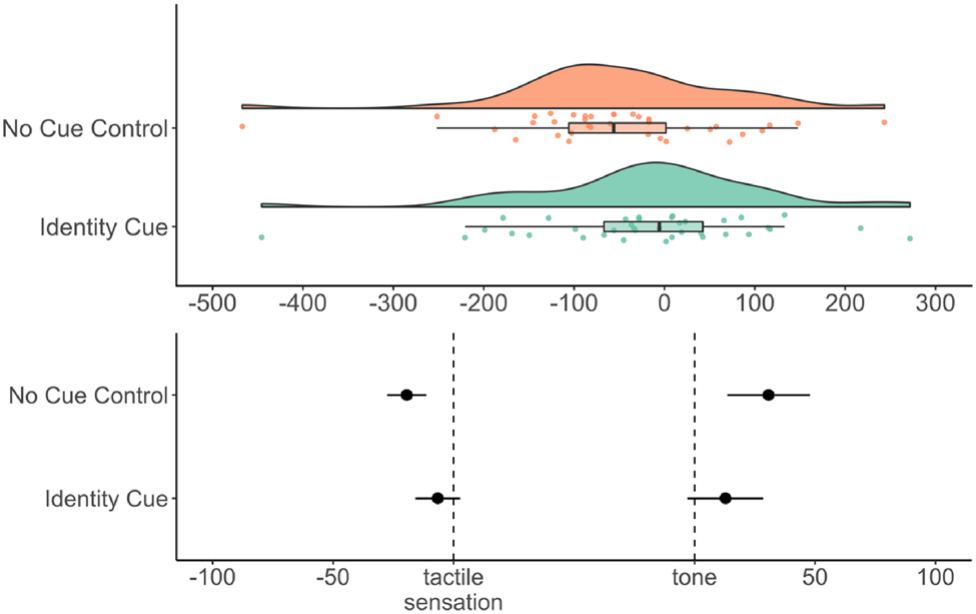
Top: Temporal binding scores in ms per condition in Experiment 2. Dots show the distribution of the individual data points, boxplot summaries use the median. Bottom: Separate temporal shifts from baseline in ms, dots denote the mean, bars represent standard errors

Overall binding in the conditions: While the temporal repulsion effect in the no cue control condition was statistically different from zero, *t*(36) =  − 2.48, *p* = 0.018, *d* = − 0. 41, 95% CI [− 0.74, − 0.07], this was not the case in the identity cue condition, *t*(36) = − 0.91, *p* = 0.367, *d* = − 0.15, 95% CI [− 0.47, 0.17]. For the distribution of the temporal binding scores in the separate conditions, see Fig. 5.

### Bayesian analysis

Overall binding: A Bayesian one-sample *t*-test of the overall binding scores against zero indicated neither evidence for temporal binding nor for temporal repulsion, BF_10_ = 0.79, 95% CrI [− 0.59, 0.05].

Influence of cue: A Bayesian paired samples *t*-test testing the hypothesis that the population mean of the no cue control condition was larger than in identity cue condition resulted in a BF_+0_ equal to 1.83, 95% CrI [− 0.63, − 0.04], indicating anecdotal evidence for the alternative hypothesis.

Overall binding in the conditions: Two separate Bayesian one-sample *t*-tests testing the null hypothesis that the population mean is equal to zero against the alternative hypothesis that the population mean is not equal to zero were conducted. For the no cue control condition, the test revealed a BF_10_ = 2.57, 95% CrI [− 0.71, − 0.05], indicating that the data was 2.5 times more likely to occur under the alternative than under the null hypothesis and therefore anecdotal evidence in favor of the alternative hypothesis. For the estimates in the identity cue condition, a BF_10_ = 0.26, 95% CrI [− 0.45, 0.18], indicating evidence for the null hypothesis.

### Experiment 3

Experiment 2 tested the robustness of our findings and investigated whether expectations about the identity of a tactile sensation facilitate temporal binding between a tactile sensation and a resulting auditory stimulus. We replicated the temporal repulsion effect and found evidence for an attenuation of the effect when the identity of the tactile sensation was explicitly known in advance. Nonetheless, prior knowledge about the identity of the tactile sensation was not sufficient to cause temporal binding.

In Experiment 3, we added another important component of intentional action, namely the temporal onset of motor action (Brass and Haggard 2008). Whereas participants in Experiment 2 were explicitly told to use the identity of the flash as a cue for which tactile stimulation (left or right) would occur, in Experiment 3, we explicitly instructed participants to anticipate the identity and temporal onset of the tactile sensation. To achieve this, the respective identity cue of the tactile sensation (left or right) flashed up in a stable rhythmic pattern, serving as a countdown for the temporal onset of the tactile sensation. This way, participants were not only informed about the identity of the sensation, but they also were able to anticipate the temporal onset of the tactile sensation If temporal control of motor movement is central to intentional action and shaping a sense of agency, further attenuation of the temporal repulsion effect and even reversal to temporal binding may emerge when participants can anticipate the temporal onset of the tactile sensation. Accordingly, to test this in Experiment 3 we compared the identity cue only condition of Experiment 2 with a new identity + temporal onset cue condition.

## Methods

### Participants and design

Forty-two^3^ (29 females) volunteers with a mean age of 24 years (*M* = 24.02, SD = 6.15), who did not take part in Experiment 1 or 2, participated in this study in exchange for course credit or monetary reimbursement. All participants provided written informed consent.

The experiment used a 2 (target: tactile vs. auditory stimulus) × 2 (type of trial: baseline vs. succession trials) × 2 (cue: identity only vs. identity + temporal onset) within-subjects design.

### Procedure

The general procedure and the experimental task resembled Experiment 2. Differences between the experiments are outlined below.

Acquisition phase: The acquisition phase was identical to Experiment 2.

Experimental trials (altered Libet clock trials): Participants completed two within-subjects conditions—an *identity cue only condition* (identical to the cue condition of Experiment 2) and an *identity* + *temporal onset cue condition*—in counterbalanced order.

Each condition consisted of the same four randomized blocks. To familiarize participants with the differences between the conditions, they completed four separate learning trials at the beginning of each condition—one of each type of block (i.e., baseline tactile, a baseline auditory, a succession tactile and a succession auditory trial in sequential order).

**Fig. 6:**
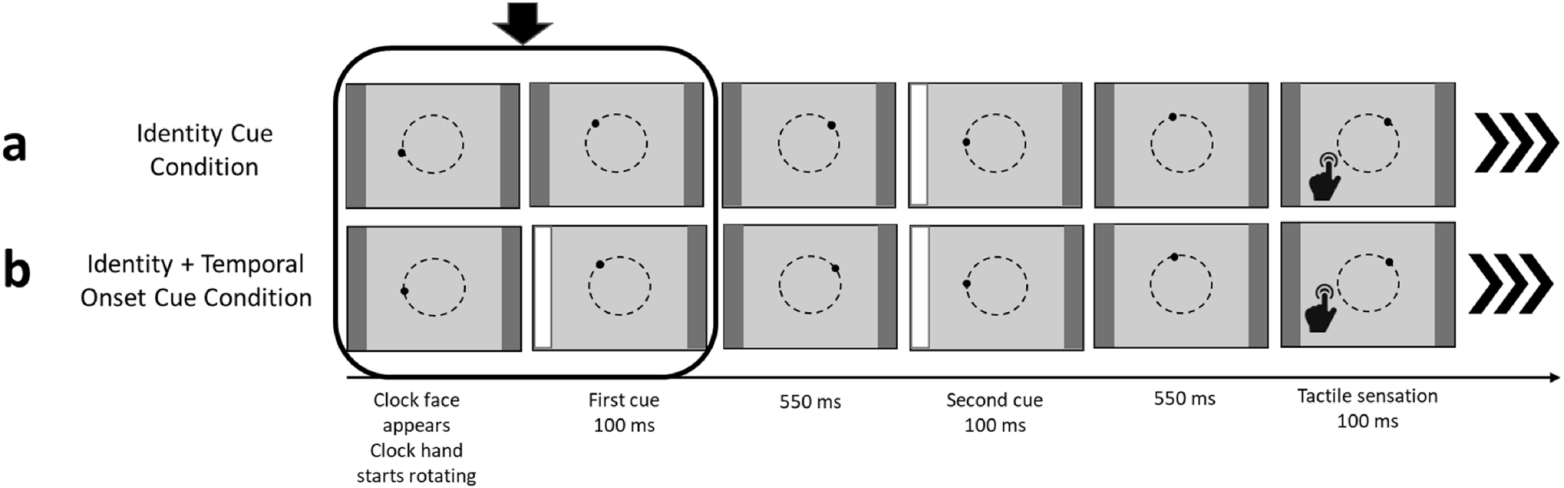
Schematic overview of the start of a trial in the two types of conditions: **a** identity cue condition (identical to Exp. 2) and **b** identity + temporal onset cue condition. In the identity + temporal onset condition, two flashes (e.g., left) occur before the tactile input (left index finger). The time window after the first and second flash is equal (550 ms), thus serving as a countdown procedure for the temporal onset of the tactile stimulation. The rectangle highlights the difference between the beginning of trials in the two conditions. The triple arrows (in bold) indicate the remaining part of a trial, which was identical to Experiments 1 and 2

The conditions differed in the cues delivered. In the *identity cue condition*, the procedure was identical to the one employed in Experiment 2. In the new *identity* + *temporal onset cue condition*, one of the columns flashed up white for 100 ms twice (e.g., left, left) before the tactile stimulation occurred. The time window after each flash was equal (550 ms), thus forming a clear rhythmic pattern. This enabled participants to use the successively presented cues as a time counter for the onset of the tactile stimulation of the index finger. Accordingly, the identity + temporal onset cue condition not only informed participants which index finger would be stimulated, but also allowed them to more carefully anticipate at which moment in time this stimulation would take place. The cues were only presented on trials in which a tactile sensation occurred (i.e., baseline tactile, succession tactile and succession auditory). Figure 6 displays the start of a trial in both conditions.

### Data analysis plan

The data of two participants was excluded as technical issues caused inadequate display of the temporal onset cues, resulting in a final sample of 40 participants.

As also done in Experiment 1 and 2, extreme judgment errors more than ± 640 ms away from the actual temporal onset were excluded as inattentiveness on those trials can be assumed (Aarts et al. 2012), resulting in the exclusion of 0.42% of all trials in Experiment 3. Moreover, the same dependent variables (i.e., perceptual shifts and overall binding scores) as in the preceding experiments were computed for each participant and condition.

We again first conducted a repeated measures ANOVA on the overall shift scores with cue as a within-subjects factor and order of the conditions as a fixed factor to assure that the order in which the two conditions were presented did not significantly affect participants’ judgment errors. We then continued with testing whether temporal repulsion or temporal binding was present in the overall sample. To that end, we conducted a two-tailed one-sample *t*-test on the overall binding scores against zero. Next, a one-tailed paired-samples *t*-test was conducted on the shift scores to test if the identity + temporal onset cue helped coherence perception (i.e., weaker temporal repulsion) as compared to the identity cue. Finally, to test whether temporal binding or temporal repulsion was present in both conditions, two-tailed one-sample *t*-tests against zero were conducted.

As in Experiment 1 and 2, we conducted parallel equivalent Bayesian analysis to assess the strength of the evidence.

## Results

Effect of order: The results of the repeated measures ANOVA on the overall shift scores with cue as a within-subjects factor and order of the conditions as a fixed factor showed no main effect for cue, *F*(1, 38) = 1.57, *p* = 0.217, *η*_*p*_^2^ = 0.04, no main effect for order, *F*(1, 38) = 3.08, *p* = 0.087, *η*_*p*_^2^ = 0.08 and no interaction between cue and order, *F*(1, 38) = 1.76, *p* = 0.193, η_p_^2^ = 0.04. Hence, the data was collapsed.

Overall binding: The results of the one-sample *t*-test against zero showed that the mean temporal repulsion effect (*M* = − 27.88, *SD* = 89.96) was not statistically significant, *t*(39) =  − 1.96, *p* = 0.057, *d* = − 0.31, 95% CI [− 0.63, 0.01].

Differences between conditions: The one-tailed paired-samples *t*-test showed that the repulsion effect in the identity cue condition (*M* = − 36.83, SD = 82.21) was stronger than the identity + temporal onset cue condition (*M* = − 18.92, SD = 111.59), but this was not significant, *t*(39) =  − 1.47, *p* = 0.077, *d*_*z*_ = − 0.18, 95% CI [− 0.43, 0.07].

**Fig. 7:**
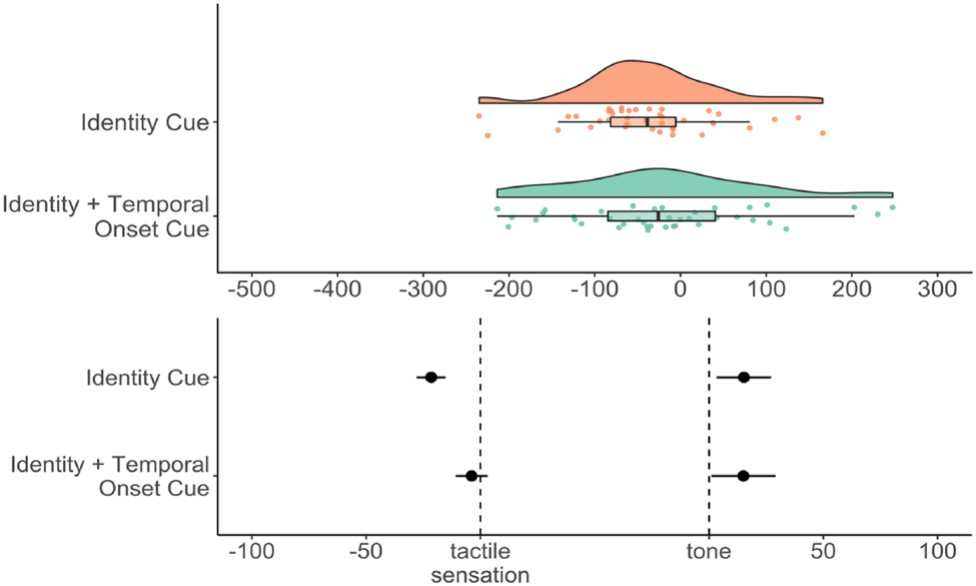
Top: Temporal binding scores in ms per condition in Experiment 3. Dots show the distribution of the individual data points, boxplot summaries use the median. Bottom: Separate temporal shifts from baseline in ms, dots denote the mean, bars represent standard errors

Overall binding in the conditions: Whereas a significant temporal repulsion effect was found in the identity cue condition, *t*(39) =  − 2.83, *p* = 0.007, *d* = − 0.45, 95% CI [− 0.77, − 0.12], the shift was not significant in the identity + temporal onset cue condition, *t*(39) =  − 1.07, *p* = 0.29, *d* = − 0.17, 95% CI [− 0.48, 0.14]. See Fig. 7 for a visualization of the temporal binding scores and shifts in the separate conditions.

### Bayesian analysis

Overall binding: A Bayesian one-sample *t*-test of the overall binding scores against the alternative hypothesis of no difference showed no evidence in favor of temporal repulsion, BF_10_ = 0.96, 95% CrI [− 0.6, 0.02].

Differences between conditions: A Bayesian paired-samples *t*-test conducted on the overall shifts scores in both conditions showed no evidence in favor of the alternative hypothesis that there was less temporal repulsion in the identity + temporal onset cue condition than in the identity cue condition over the null hypothesis of no difference, BF_+0_ = 0.83, 95% CrI [− 0.52, − 0.02].

Overall binding in the conditions: Separate Bayesian one-sample *t*-tests on the overall shift scores testing the hypothesis that the population mean does not equal zero were conducted. For the identity cue condition, a BF_10_ of 5.37, 95% CrI [− 0.73, − 0.1] and thus substantial evidence for the hypothesis that the population mean differed from zero was found. In the identity + temporal onset cue condition, there was no evidence for a deviation of the population mean from zero, BF_10_ = 0.29, 95% CrI [− 0.45, 0.14].

## General discussion

The present research addressed the question of whether intentional binding as a reflection of the sense of agency occurs in situations where an agent does not engage in motor action. As noted in the introduction, previous research suggests that temporal binding emerges even when people do not actively engage in goal-directed action (Desantis et al. 2011; Dogge et al. 2012; Kirsch et al. 2019; Suzuki et al. 2019; Vastano et al. 2018).

Here, we further explored the processes involved in temporal binding by examining the role of self-related bodily sensations in temporal binding of such sensations and a resulting effect in the full absence of motor action. Specifically, we delivered tactile input to the fingertip as a haptic sensation that usually accompanies a finger press on a key to cause an effect (e.g., an auditory stimulus). If temporal binding occurred, this would demonstrate that, in principle, people could experience agency over sensory events that do not result from actual motor movement. Instead of observing temporal binding, we established a robust temporal repulsion effect between a tactile sensation on the fingertip and the auditory effect that resulted from it, suggesting a dramatic disruption of the sense of agency.

Temporal repulsion has been observed before between two stimuli (Antusch et al. 2020; Desantis et al. 2012; Haggard et al. 2002a), and between TMS-induced finger movements and effects (Haggard et al. 2002b). The occurrence of temporal repulsion suggests that a tactile sensation and an auditory effect are separated from each other instead of being integrated into a coherent representation. Although stimuli of different modalities are often bound together in natural perception, this tendency is diminished if the combination is less naturalistic (see Antusch et al. 2020), which may explain the absence of binding and even repulsion. Although speculative, repulsion may therefore help to perceive events that occur almost instantaneously as separate, if there is no clear common underlying natural cause.

As previous research on the sense of agency suggests, intentionality and corresponding motor actions are an important amplifier of temporal binding (Antusch et al. 2019; Moore and Ohbi 2012; Tanaka and Kawabata 2019). Intentional action relies on a sensorimotor control model that is generally assumed to capture the identity and temporal onset of intentional action and its sensory consequences (Blakemore et al. 2002; Haggard 2017), thus linking them together in the perception of the agent. However, a similar mechanism is absent during passive tactile sensation, even though such sensations are usually part of intentional movement itself. Accordingly, intentional action and passive tactile stimulation differ in the expectation of which stimulus sensation will occur when.

Indeed, our findings indicated that repulsion between a sheer tactile stimulation and an auditory effect was partly corrected when participants were able to anticipate which tactile sensation (on the left or right index finger) would occur and when it would occur. That is, participants perceived more coherence between the tactile sensation and its effect and experienced them as being more temporally related when they were explicitly encouraged to predict the identity and temporal onset of the haptic sensation. This concurs with other research arguing that predictive processes are the primary source that links events together in awareness (Eagleman and Holcombe 2002; see also Tanaka et al. 2019). Importantly, although information of the identity and anticipation of temporal onset of the haptic sensation improved perceptual binding between tactile sensation and auditory effect, it did not cause temporal binding.

An important question arising from our findings is why we did not find temporal repulsion to be reversed into temporal binding when crucial agency information about the identity and temporal onset was available, especially in light of temporal binding effects obtained in previous work on observation of movement (Suzuki et al. 2019; Vastano et al. 2018). Although we do not have a conclusive answer, we offer two possible explanations and suggestions for further research. First, the relation between the haptic event (left or right finger stimulation) and the distinct effect (i.e., a high or low tone) was arbitrary and had to be learned. While this is the case for most intentional binding studies, this does not constitute a natural setting and may be the reason temporal repulsion was observed. It may be the case that a natural overlap between the properties of haptic events and outcomes (e.g., left finger stimulation leads to a tone in the left ear) may reduce repulsion more strongly or even create binding. Note, though, that such a manipulation would not rely on a learned relation between actions and outcomes, but on a universal or overlearned relation between the two (Dogge et al. 2019a, b).

A second explanation would be that motor simulation is a prerequisite for binding to occur. Perhaps observation or mechanical induction of movement allows for covert activation of motor programs and resulting efference copies that are compared with the resulting effect to create binding (Blakemore et al. 2002). If correct, this would suggest that motor simulation did not occur—or at least not at the right time—neither in the experiment reported here, nor in studies where finger movements were induced by TMS (Haggard et al. 2002b). As this explanation is entirely speculative, further research would be needed to test this hypothesis.

To conclude, we observed that the time-interval between a tactile sensation and an external sensory consequence is subjectively magnified in the absence of motor action, showing weak perceptual coherence between haptic sensations and auditory effects. Our findings suggest that temporal repulsion does not easily reverse into binding that is commonly associated with intentional action—even when a person can predict the identity and temporal onset of body sensations and effects that will occur. Therefore, while our findings point to the importance of expectations for integrating tactile sensations and external effects, intentionality seems to have a special status in binding action and effect together and thus might be crucial in shaping a sense of agency and experiences of control when interacting with the external world.

## Supplementary Information

Below is the link to the electronic supplementary material.

Supplementary file1 (DOCX 698 KB)

## Footnotes

1 As to the best of our knowledge, no literature testing a comparable effect in a tactile-auditory stimulus-based paradigm was available at the point of data collection. Hence, we decided to treat each experiment as a new data point and calculated the sample size on the basis of a moderate effect. We used G*Power 3.1 (Faul et al. 2007) with the following parameters: *f* = .25, *α* = .05, power = .80, nonsphericity correction *ε* = 1 and *r* = .5 to determine the sample size in an ANOVA: Repeated measures within (2) factors. This yielded an estimated sample size of 24 participants. Based on lab capacities, we sampled three extra participants.

2 Experiment 1 yielded a repulsion effect that was fairly strong. However, we had no hypothesis about the cue effect size. Therefore, for Experiment 2 we again calculated the sample size according to the following parameters: *f* = .25, *α* = .05, power = .80, nonsphericity correction *ε* = 1 and *r* = .5 to determine the sample size in an ANOVA: Repeated measures within (3) factors, yielding a sample size of 16 participants. To avoid power issues, we opted for a more conservative (doubled) sample size of at least *n* = 32.

3 The sample size of Experiment 3 was estimated using the following parameters: *f* = .25, *α* = .05, power = .80, nonsphericity correction *ε* = 1 and *r* = .5 to determine the sample size in an ANOVA: Repeated measures within (3) factors yielded an estimated sample size of 16 participants. We again doubled the sample size and aimed to recruit a minimum of 32 participants.
