# Supplementary material for: Studying the sense of agency in the absence of motor movement: an investigation into temporal binding of tactile sensations and auditory effects
Source: Exp Brain Res. 2021 Apr 7;239(6):1795–806. doi: 10.1007/s00221-021-06087-8 (PMC8277642; doi:10.1007/s00221-021-06087-8)

Supplementary Materials

JASP Output of the Robustness Check concerning the Results reported for the Bayesian Analysis for Experiment 1

Figure S1. Robustness check Experiment 1


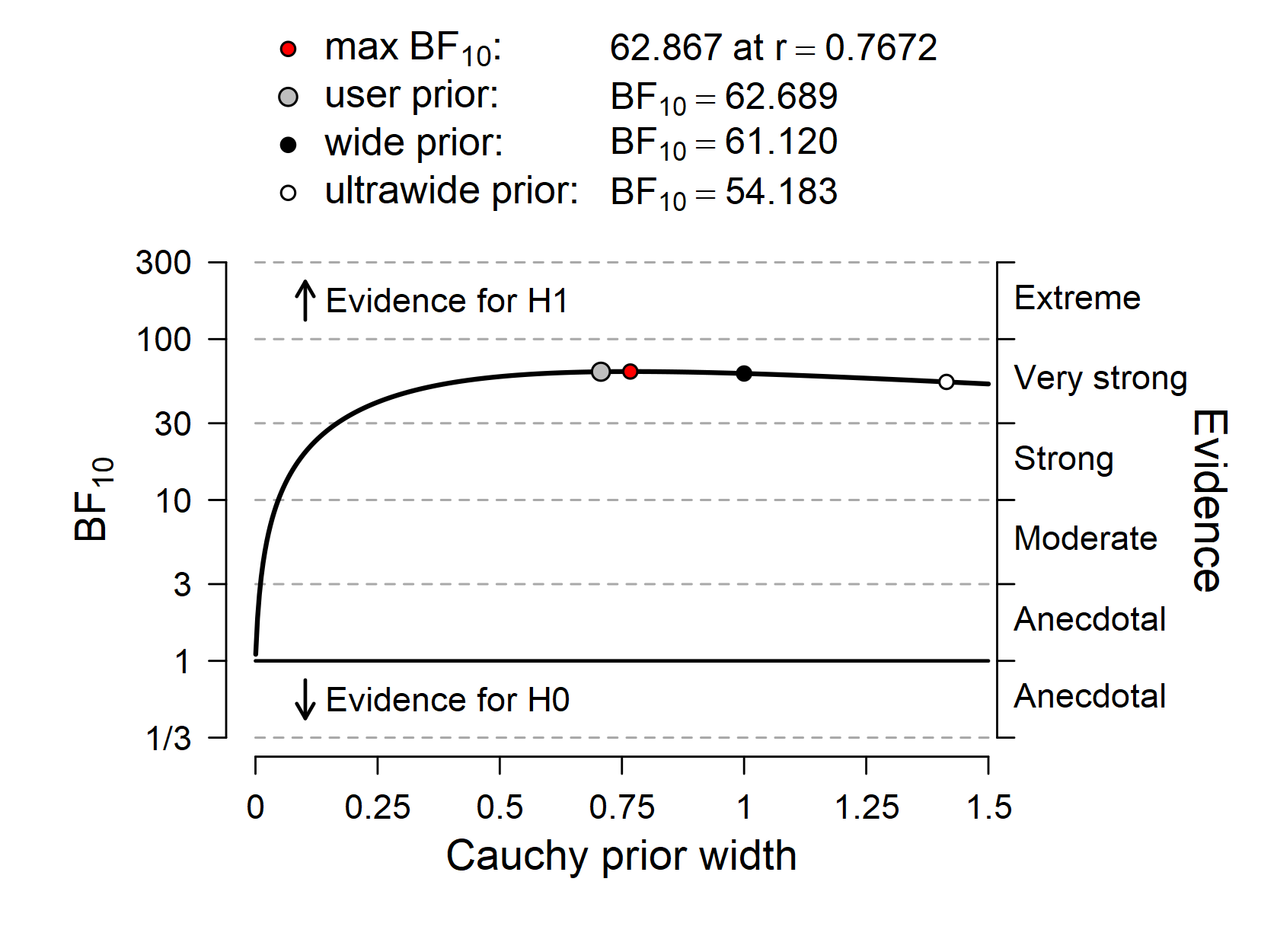


Supplementary Materials

JASP Output of the Robustness Check concerning the Results reported for the Bayesian Analysis for Experiment 2

Figure S2. Robustness check Experiment 2 testing overall binding against zero


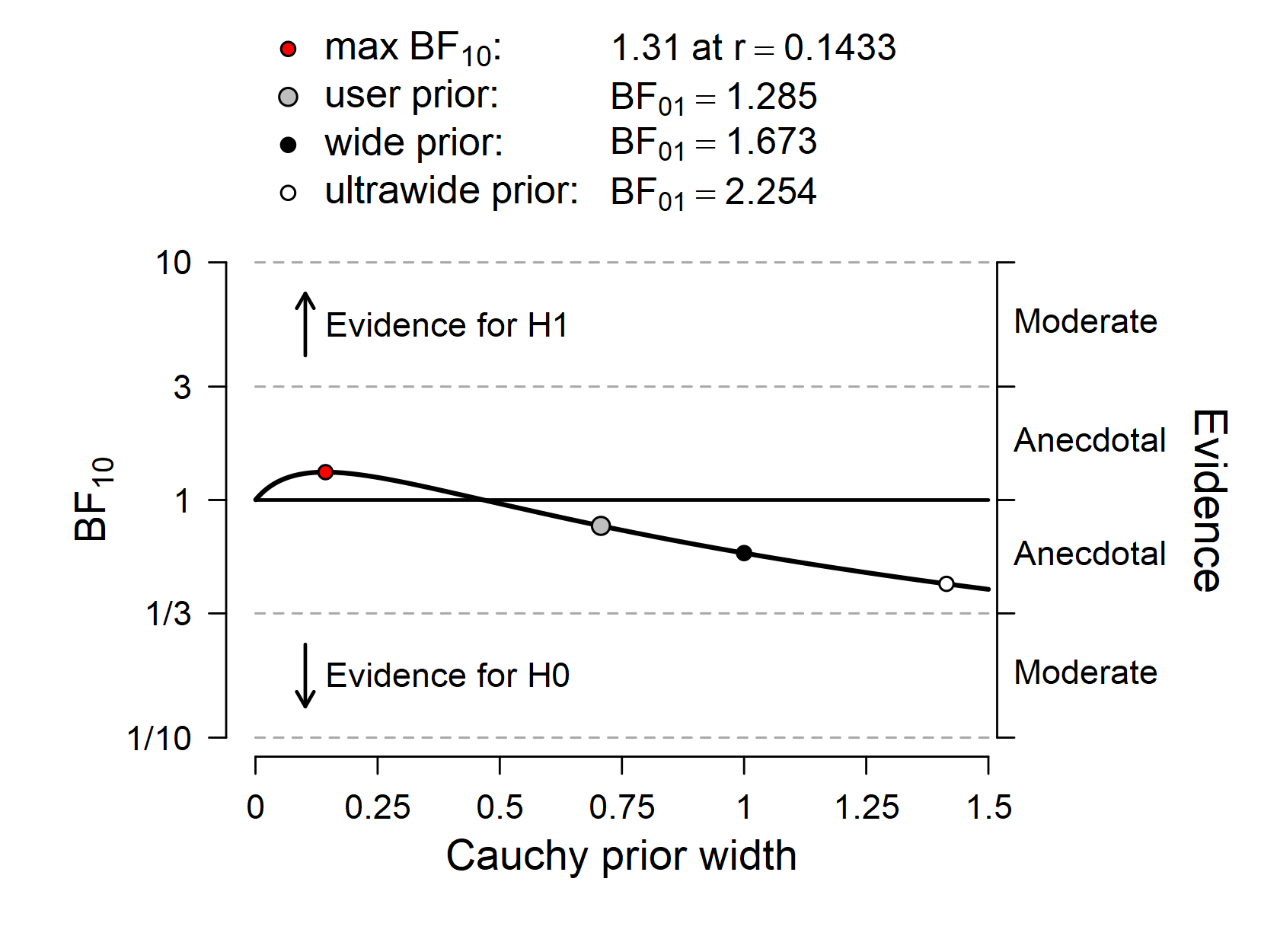


Figure S3. Robustness check Experiment 2 testing the difference between conditions


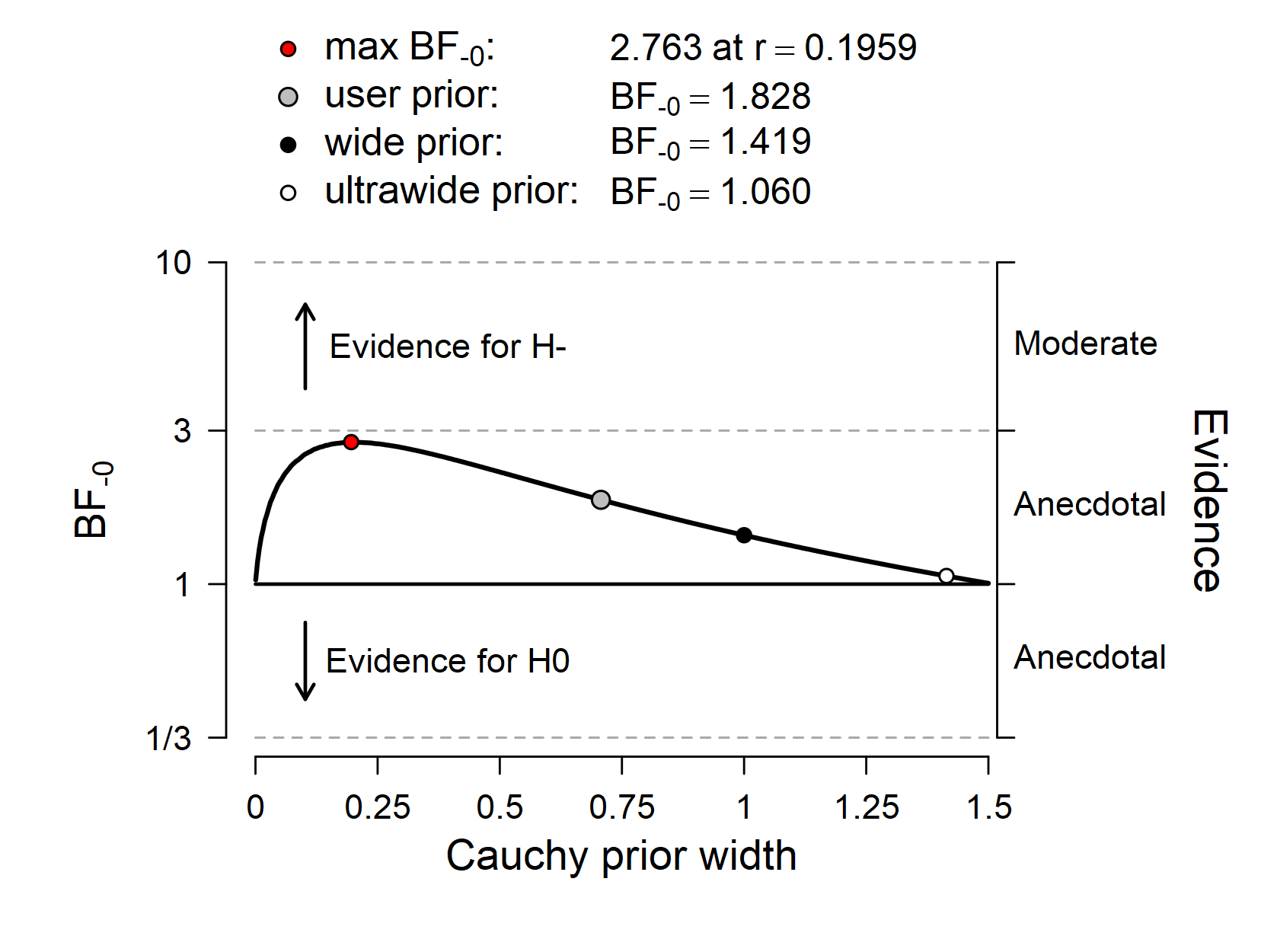


Supplementary Materials

JASP Output of the Robustness Check concerning the Results reported for the Bayesian Analysis for Experiment 2

Figure S4. Robustness check Experiment 2 testing temporal binding score against zero (no cue control condition)


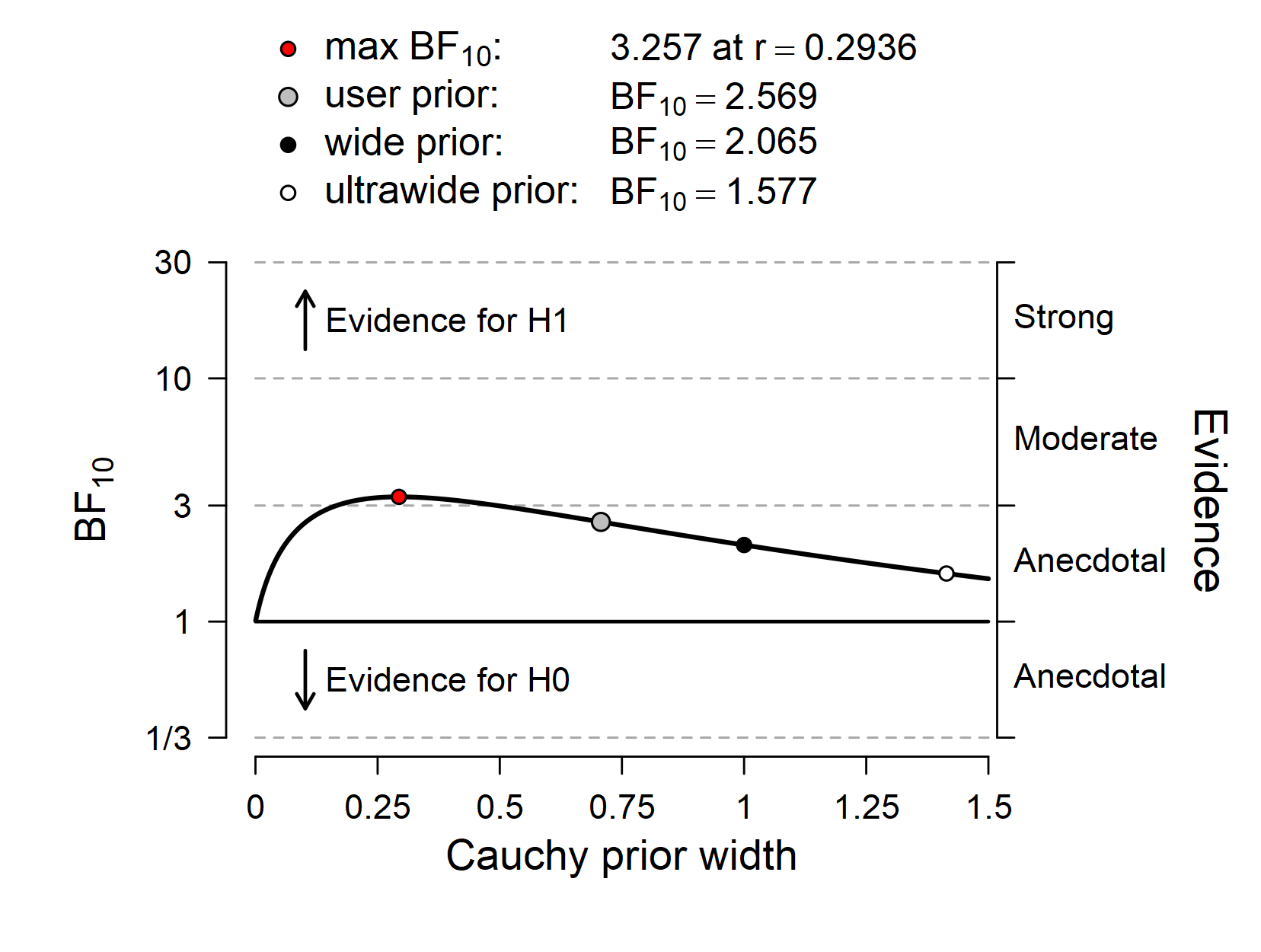


Figure S5. Robustness check Experiment 2 testing temporal binding score against zero (cue condition)


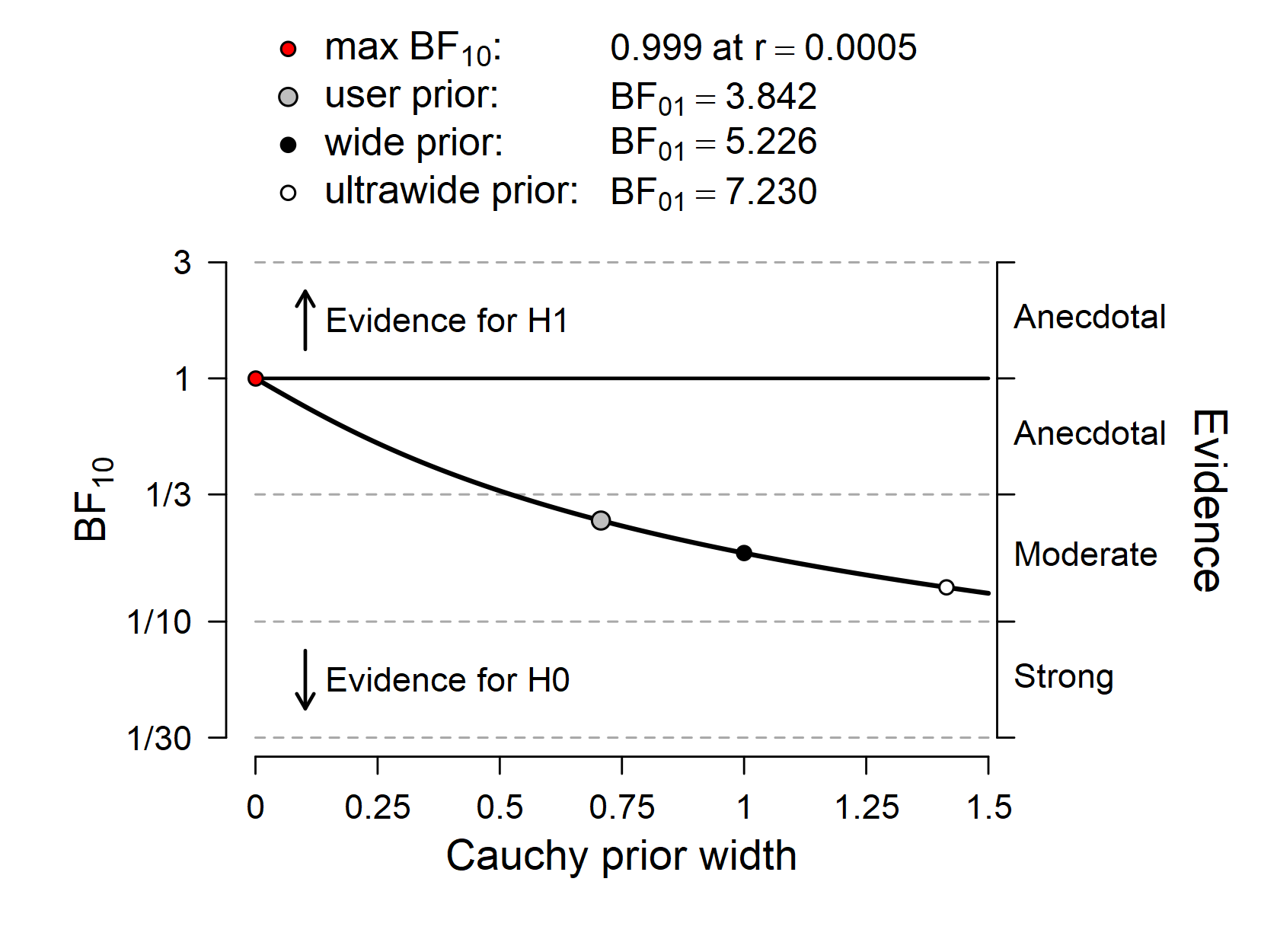


Supplementary Materials

JASP Output of the Robustness Check concerning the Results reported for the Bayesian Analysis for Experiment 3

Figure S6. Robustness check Experiment 3 testing overall binding against zero


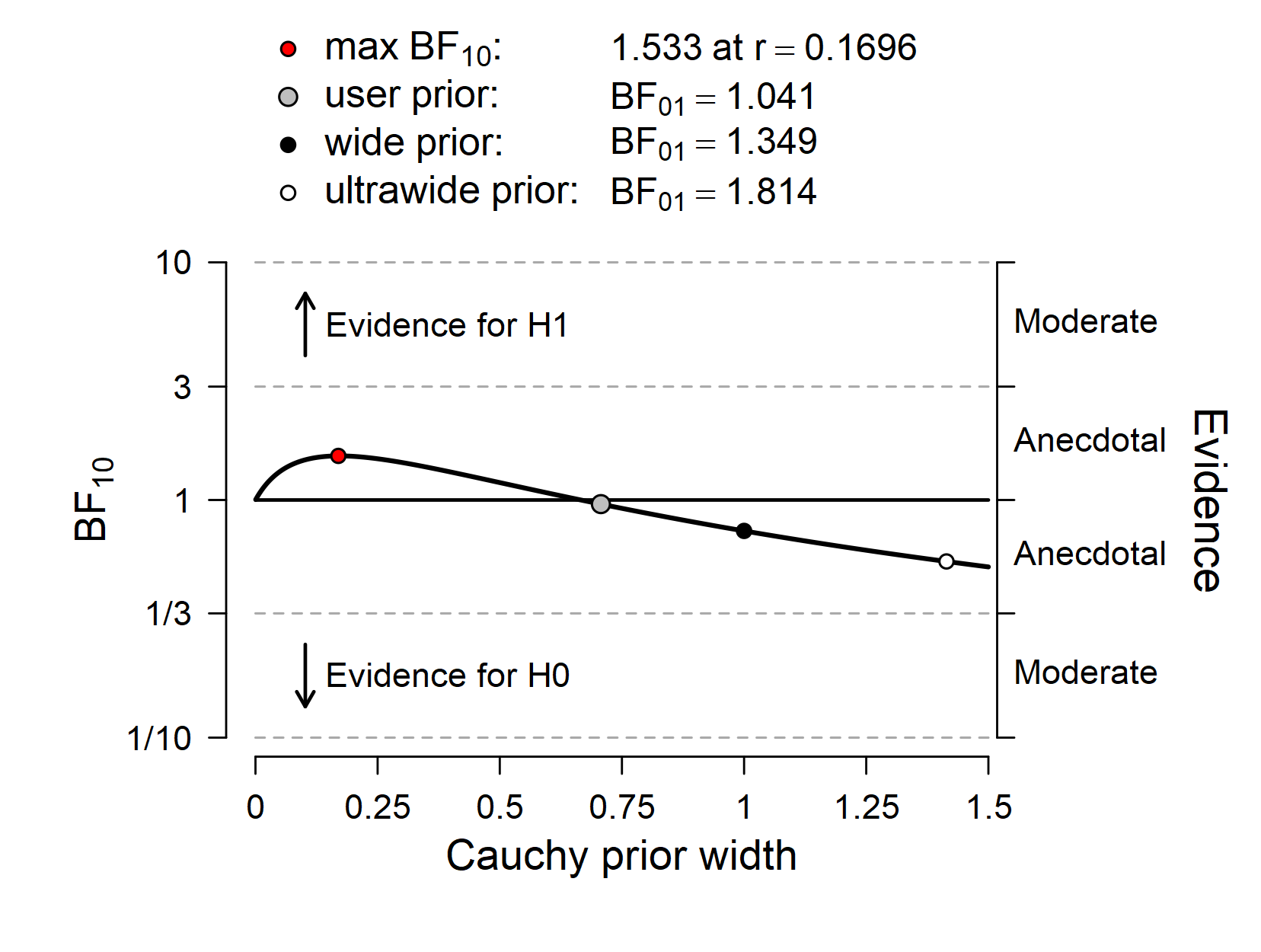


Figure S7. Robustness check Experiment 3 testing the difference between conditions


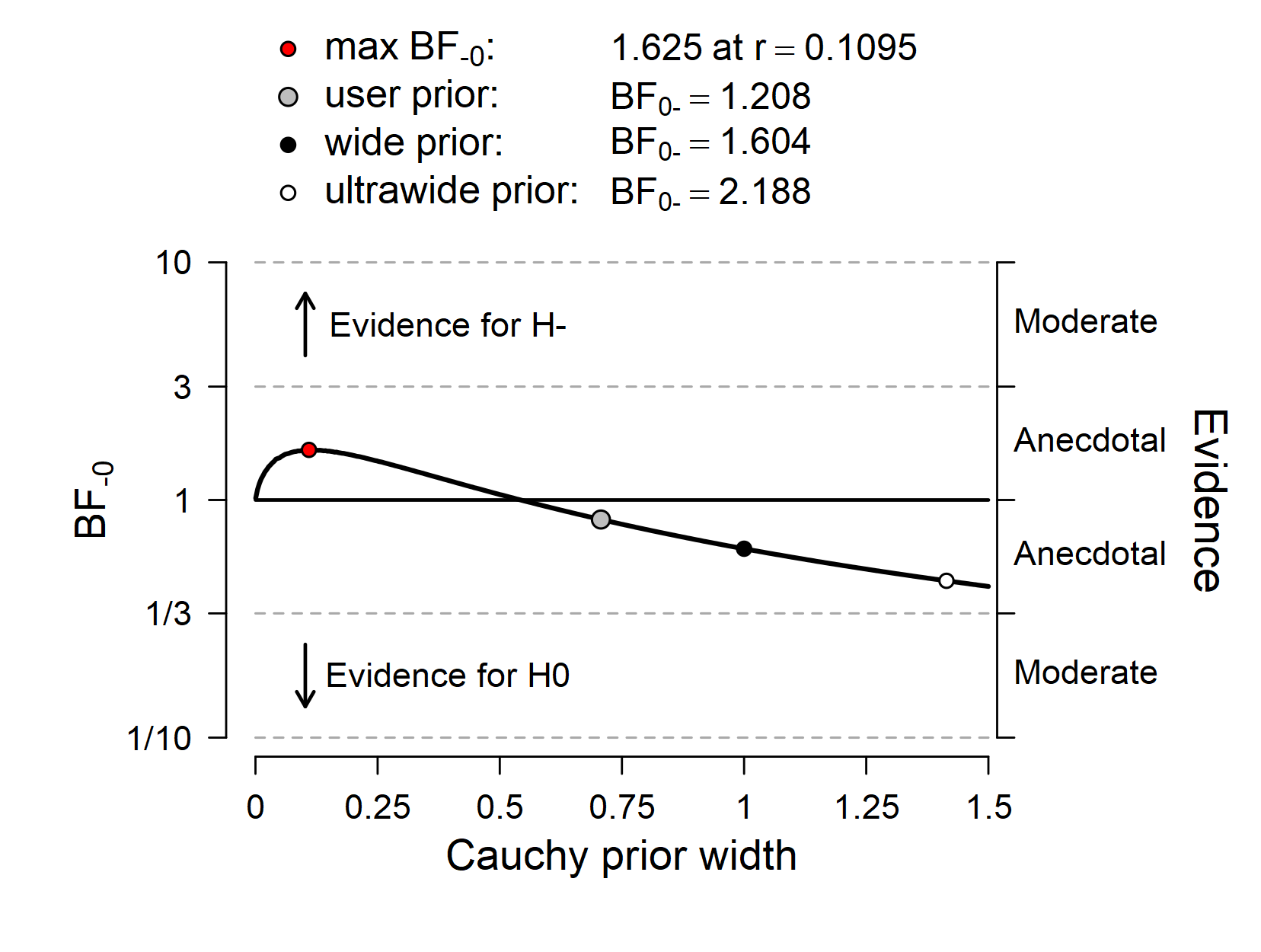


Supplementary Materials

JASP Output of the Robustness Check concerning the Results reported for the Bayesian Analysis for Experiment 3

Figure S8. Robustness check Experiment 3 testing temporal binding score against zero (direction cue condition)


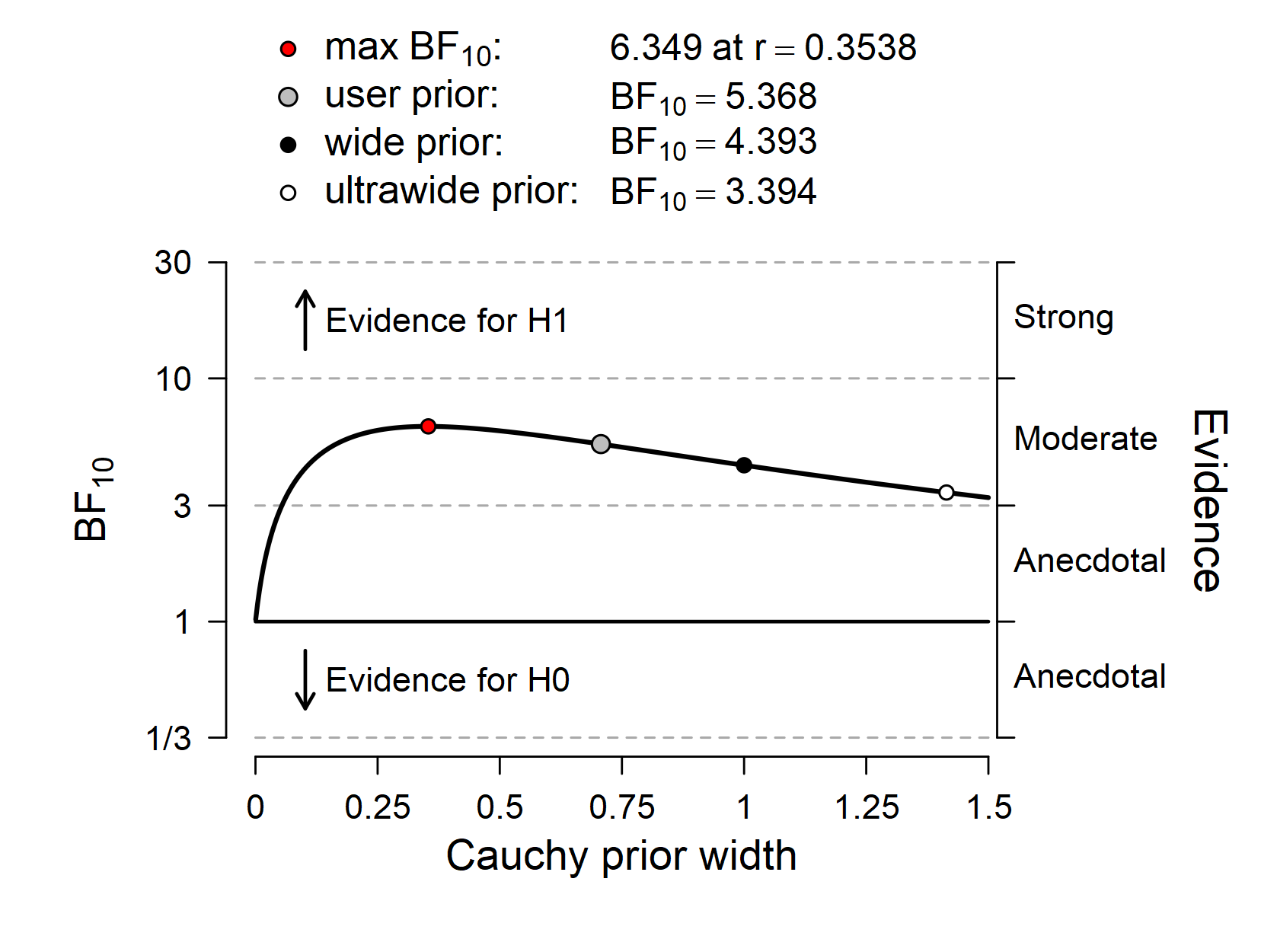


Figure S9. Robustness check Experiment 3 testing temporal binding score against zero (direction + countdown cue condition)


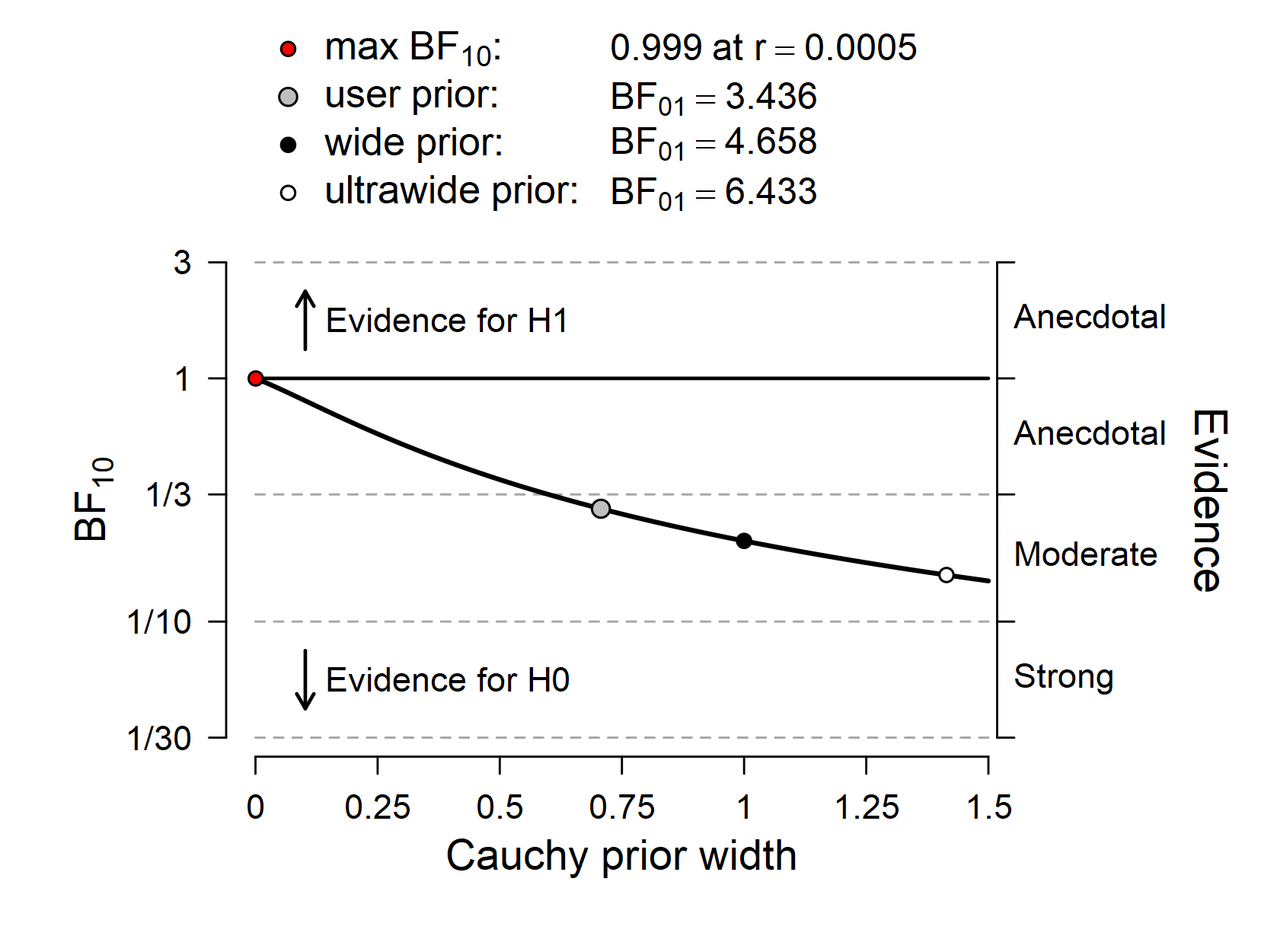

Supplement: Supplementary file 1 — Supplementary file1 (DOCX 698 KB) [file 221_2021_6087_MOESM1_ESM.docx]
